# “It’s messy and it’s massive”: How has the open science debate developed in the post-COVID era?

**DOI:** 10.12688/f1000research.162577.2

**Published:** 2025-08-04

**Authors:** Melanie T Benson Marshall, Stephen Pinfield, Pamela Abbott, Andrew Cox, Juan Pablo Alperin, Natascha Chtena, Alice Fleerackers

**Affiliations:** 1 The University of Sheffield Information School, Sheffield, England, UK; 2 Simon Fraser University Publishing Program, Vancouver, British Columbia, Canada; 3 University of Amsterdam Department of Media studies, Amsterdam, North Holland, The Netherlands

**Keywords:** Open science, open access, preprints, COVID-19, science communication, scientific culture

## Abstract

The COVID-19 pandemic accelerated the global adoption of open science (OS) practices. However, as the pandemic subsides, the debate around OS continues to evolve. This study investigates how the pandemic has shaped the OS discourse and identifies key issues and challenges. Interviews were conducted with influential actors across the research and publishing communities. The findings show that while many areas of debate remained constant, the ways in which they were discussed exposed underlying systemic challenges, which must be addressed if OS is to progress. These issues included the scope and definition of OS; regional variations in its implementation; the relationship between OS and fundamental questions of the purpose and practice of science; and the need to reform incentives and reward structures within research systems. A more complex understanding of OS is required, which takes into account the importance of equity and diversity and the challenges of implementing OS in different cultural and geographical contexts. The study emphasises the importance of shifting scientific culture to prioritise values such as quality, integrity, and openness, and reforming rewards structures to incentivise open practices.

## 1. Introduction

The COVID-19 pandemic gave rise to an intensified debate about open science (OS – the “global movement that aims to make scientific research and its outcomes freely accessible to everyone” ( Center for Open Science, n.d.)) ( Benson Marshall et al., 2024). Some advocates of OS argued that the pandemic was a ‘stress test’ of the value of OS and would act as a ‘catalyst’ for change involving more widespread adoption of open practices in the global scientific community. In contrast, critics of open practices expressed scepticism about approaches such as preprinting, which became more widespread during the medical emergency, and voiced concerns about the quality of scientific work made publicly available, linking it to the ‘infodemic’ that accompanied the pandemic. Now that the emergency of the pandemic has passed ( World Health Organization, 2023a), we have the opportunity to take stock of how the debate about OS has evolved, and to investigate the extent to which the hopes of its advocates and the fears of its critics, voiced in debates at the height of the pandemic, have been realised. In this paper, we therefore address the question: how has the COVID-19 pandemic affected the debate on OS?

In a previous study carried out in 2022 and 2023 ( Benson Marshall et al., 2024), we examined the debate about the relationship between OS and the pandemic based on publications in blogs, newspapers, magazines, and professional and peer-reviewed journals from the height of the pandemic (December 2019 to December 2022). We now build on that previous research through interviews with a diverse range of expert participants in research and publishing roles whose work relates to OS. We investigate how the debate on OS has further developed in the emerging post-pandemic period, and the extent to which COVID-19 has changed the nature of the debate. We also aim to establish which aspects of debates about OS seem to have gained traction among these influential people following what we were able to observe in writings during the pandemic, with its focus on issues such as data sharing and preprinting, and its concerns around quality and misinformation. It is important to note that our aim is not to provide an exhaustive survey of all discussions encompassed by the broad ‘open science’ umbrella, but rather to illuminate the specific points of contention and consensus in relation to the experience of the pandemic identified by those directly involved in the practices we examined. We draw out from the interviews a set of fundamental systemic issues that may be seen to underlie debates about OS, and which need to be understood in addressing some of the main challenges associated with openness. From these we present conclusions about the impact of the pandemic on the OS debate and possible trajectories for OS in the future.

## 2. Background and Literature Review

The COVID-19 pandemic led to a rapid increase in scientific inquiry and collaboration to address urgent health and social problems ( Harper et al., 2020; Jit et al., 2021). OS became an increasingly high-profile part of this work ( Ala-Kyyny, 2020; OECD, 2020). The crisis was seen by many as emphasising the critical role of OS in facilitating rapid dissemination and accessibility of vital research findings ( Science Europe, 2022), and serving as a catalyst for increased adoption of open practices ( Fraser et al., 2021; Lane & Lifshitz-Assaf, 2022). This focus on OS also highlighted its potential to advance scientific progress and impact public health policy ( Besançon et al., 2021; Horby, 2022; Taschwer, 2022).

Debates about the various facets of OS are not new, of course. They have been ongoing since at least the early 2000s, although not always using the label of ‘open science’, which has been widely adopted more recently ( Miedema, 2022; Suber, 2012; Willinsky, 2003, 2006). While adoption of OS practices has increased, aspects of it remain controversial, including questions over funding and business models, infrastructure development, researcher incentives, and global equity in research systems ( Miedema, 2022; Pinfield, 2024; Pinfield et al., 2020; Simard et al., 2022). While OS facilitated successes in the pandemic, such as rapid genome sequencing and vaccine development ( Chen et al., 2022), global inequalities in scientific and medical capacity were also brought into the spotlight ( Benach, 2021; Bhaskar et al., 2020; Jensen et al., 2021). This drew greater attention to these debates and their implications for what is increasingly seen as a global research system ( Marginson, 2022), but should also be acknowledged as a collection of smaller, interconnected systems.

The pandemic also heightened political dimensions of science in general and OS in particular. Scientists became more directly involved in advising politicians and shaping public policy, using the latest science, including open outputs ( Ball, 2021; Colman et al., 2021; Joubert et al., 2023). OS was encouraged by many governments and research agencies as a way of accelerating the scientific response to the medical emergency. The Wellcome Trust, based in the UK, coordinated the creation of an influential statement in early 2020 committing its 160 international signatory organisations to more open practices ( Wellcome, 2020). Other major international policy initiatives designed to further OS beyond the COVID-19 context were also launched, notably the memorandum on ‘Ensuring Free, Immediate, and Equitable Access to Federally Funded Research’ issued by Dr Alondra Nelson on behalf of the Office of Science and Technology Policy in the USA ( Nelson, 2022), and UNESCO’s (2021) ‘Recommendation on Open Science’. The second of these initiatives brought to the international stage an expanded understanding of OS that encompasses engagement with diverse actors and knowledge systems in addition to concerns about access to research knowledge and infrastructure. This broader understanding of OS underscores the importance of communicating scientific knowledge to a wider range of people and groups, encompassing what the UNESCO Recommendation refers to as “societal actors beyond the traditional scientific community” ( UNESCO, 2021, p. 8). The Recommendation is becoming a significant reference point for understanding and implementing OS principles; it appears to have influenced how openness is conceptualised and discussed in policy documents, particularly in Europe and Latin America ( Chtena et al., 2023).

While the concept of OS has gained global traction, its conceptualisation and adoption vary across regions and cultural contexts ( de Oliveira et al., 2021; Moskovkin et al., 2021; Simard et al., 2022). For example, emphasis on specific aspects of OS differs, and the pace of adoption and challenges faced in implementing OS practices also varies considerably. The COVID-19 pandemic may have accelerated the global adoption of OS principles and practices in some respects ( Kadakia et al., 2021), but the impact of this has been uneven. This illustrates that OS is not a single universally applicable model, but rather a contested and evolving set of practices—shaped by national policy environments, infrastructural conditions, disciplinary norms, and positionalities within the global knowledge economy. Prior research has often overlooked these geographic and cultural differences, identifying a need for a more nuanced understanding of how OS is perceived and implemented in different contexts.

A particular challenge lies in navigating the relationships between scientific and Indigenous knowledge systems, with a closer connection between them explicitly seen as a core part of OS in the UNESCO Recommendation. Indigenous knowledges are extremely diverse, but many share some core characteristics. They are often community-based and developed and shared collectively rather than in the individualistic fashion of Western science ( Aikenhead & Ogawa, 2007). They also tend to be explicitly place-specific rather than attempting to be generalisable or applicable to different contexts ( Aikenhead & Ogawa, 2007). The oral and metaphorical nature of many Indigenous knowledges means that they are transmitted through specific cultural and Indigenous information exchange mechanisms, often by elders or specialists such as healers, and at specific times or occasions ( First Nations Information Governance Centre, 2024). Only a few aspects of Indigenous knowledge are formalised or codified in ways that are typically used by Western science ( Semali et al., 1999). While Western science is typically based on empirical observation and experimentation, Indigenous knowledges are often deeply rooted in the cultural, historical, and environmental contexts of their people and may be based on spiritual beliefs and traditions ( Semali et al., 1999). In many Indigenous cultures such knowledge is sacred or confidential, and is not something that should be shared outside of very specific contexts, communities, or even seasons ( First Nations Information Governance Centre, 2024). Such characteristics conflict with the desire for openness and transparency ( Ravindran, 2024) and therefore create challenges for their integration with OS perspectives.

In previous work, we examined the international debate on OS that took place during the pandemic through analysis of key articles, editorials, blogs and thought pieces ( Benson Marshall et al., 2024). The research found that the pandemic had reinforced the perceived benefits of OS, in part because OS was seen to have in turn played a crucial role in the pandemic response, demonstrating a clear ‘line of sight’ between open practices and benefits for both science and society. We observed that while the core arguments for OS had remained consistent, the new context in which they were set had given them a renewed urgency. Additionally, the focus of debates had shifted during the pandemic: business models and critical perspectives on OS received less attention than might have been expected, while discussions of open data (OD), preprinting, information and research quality, and issues around misinformation became more prominent. There was also a growing emphasis on reframing the conceptualisation of OS to better connect with society and address concerns regarding equity—aims advanced and supported by the UNESCO Recommendation.

## 3. Methodology

This study was designed to enrich and extend the findings of our earlier study ( Benson Marshall et al., 2024) through interviews with experts involved in research systems in general and OS in particular. Interviews focused on their perceptions of the relationship between the pandemic and OS, as we invited them to reflect on how this relationship will impact the future of OS in the aftermath of the public health emergency.

### 3.1 Data collection

Interviews with 33 individuals were conducted between November 2023 and May 2024—a period following the WHO’s declaration of the end of the public health emergency on May 5, 2023 ( World Health Organization, 2023b). A heterogeneous purposive sampling approach was used, aiming to create a diverse set of interviewees in terms of professional role, geographical location, and gender. Roles targeted included policymakers, platform and service providers, publishers, journalists, researchers, and librarians, all of whom had a connection with OS policy and activity. In practice, many participants could be assigned to more than one of these categories, as they performed multiple roles and were able to speak from different perspectives within a single interview. The sample was not intended to be fully representative of the diversity of roles and perspectives on OS, but was selected to provide coverage of the key roles and main arguments, building on previous work ( Benson Marshall et al., 2024) and addressing the objectives of the study. It represents the perspectives of those engaged in the OS debate, but it may not fully capture the views of all actors, particularly those who may hold less defined views on OS, or those who prefer to remain silent in order to protect their interests. The approach taken corresponds to the approach often known as “elite” or “expert” interviews, in which people with influence, status, or expertise in a certain domain are invited to participate ( Niu, 2024) using what is known as “expert sampling” ( Etikan et al., 2015).

A list of potential participants was compiled from authors of previously examined prominent publications about OS during the pandemic and from contacts known to the research team or their contacts, then augmented using snowball sampling from participants and their contacts ( Vlckova et al., 2024). Certain groups, actors, and regions were targeted either because their contribution to the text corpus analysed in our prior work was deemed highly prominent or significant, or because their voice was missing from that debate ( Benson Marshall et al., 2024). The final sample comprised 19 male and 14 female interviewees. The geographical makeup included 12 participants from Europe, eight from North America, four from Asia, three from Africa, three from South America, and one from Australasia; two participants were based in Europe but worked in the contexts of Africa and the Middle East, upon which their interviews largely focused. In terms of roles, 14 participants were involved in publishing, nine in conducting research in various fields including metascience, five in policymaking and funding, five in platform or service provision, four in journalism or science communication, and three in libraries (as noted, interviewees often performed multiple roles within the OS space, and this intersection was seen as a valuable contribution).

Participants were approached by email and provided with information about the nature of the study and the wider project, as well as the background and research interests of the interviewer. Written informed consent was given for participation, and also for publication by those who gave permission for their details to be included. Before their interviews, participants were offered three options for anonymity: (1) permission for their name and organisation to be shared in research outputs; (2) permission for their organisation but not for their name to be shared in research outputs; (3) no permission for their name or organisation to be shared. We respect participants’ wishes when reporting the findings below; this means that even for partially identified participants, some views and quotes are not linked to individuals, in line with their requests. Participants who did not want to be named are referred to using a participant code. The study design was given approval within the University of Sheffield Research Ethics regulations (application 056970).

Interviews were conducted by the first named author, a female postdoctoral researcher who is experienced in qualitative interviewing. The interviews were held in English and lasted between 30-75 minutes. They were semi-structured, with a core list of questions plus the opportunity to explore individual topics in more detail as necessitated by participants’ role and area of interest; the interview guide addressed areas of interest that were informed by previous work ( Benson Marshall et al., 2024). Questions addressed changes seen in OS practices during the pandemic; the impact of OS on the pandemic response, and vice versa; the role of science communication; the expanded definition of OS as framed by the UNESCO Recommendation; and future trends and critical issues for both OS and science in general. The interview guide is available at: [ Interview guide - for publication].

### 3.2 Data analysis

Interviews were conducted and recorded via video conferencing, transcribed using transcription software, then manually checked and edited for accuracy. Transcripts were returned to participants where requested for comment and/or correction. Notes were made after each interview on prominent themes. A qualitative inductive content analysis of the transcripts was conducted in NVivo 1.7.1, using thematic analysis approaches ( Terry et al., 2017); this analysis was primarily guided by the overarching research questions, but also significantly informed by the findings from Benson Marshall et al. (2024). The first step involved familiarisation with the data, which entailed re-reading all transcripts and taking preliminary notes on potential themes.

The coding process began with open coding, where initial codes were generated inductively. This involved a line-by-line approach to the transcripts, identifying and labelling concepts, ideas, and experiences. Both semantic (identifying explicit meanings) and latent coding (interpreting underlying meanings and assumptions) were employed. The initial codes were primarily generated by one researcher (MBM) and then validated by other members of the research team through their independent reading of the source material. A small group of four team members initiated the coding process by all independently coding the same pieces of data. They then met to discuss their findings and agree upon a consistent approach for the subsequent coding. This process was highly iterative, with regular discussions among the research team regarding the developing codebook. These discussions helped refine code definitions and ensure consistency. This collaborative effort resulted in the generation of 120 distinct codes. Following the initial coding, codes were systematically sorted and collated, and themes reviewed and refined. While we partly looked to confirm or deny findings from Benson Marshall et al. (2024), our primary focus shifted to identifying and describing a series of underlying, broader themes that emerged organically from the current data. We intentionally did not aim to duplicate the more close, exhaustive analysis of the first paper. Instead, our analysis was specifically focused on these new and broader themes that arose, rather than reporting on every single theme noted.

To further enhance the rigor of the analysis, other team members independently read the transcripts to discern emerging themes and findings, making detailed notes on each transcript. These emergent themes and findings were then extensively discussed among the research team. These discussions were crucial in confirming interpretations and enhancing the dependability of the results.

## 4. Findings and Discussion

### 4.1 Focuses of debate

Our interviews confirmed that many of the issues reported in Benson Marshall et al. (2024) as focuses of the OS debates during the pandemic were still seen as crucial in the emerging post-pandemic context. They included the value of preprinting and data sharing, and the influence of the pandemic in driving OS practice and policy development—issues that participants were now able to discuss in a reflective way, with some distance from the emergency. It is notable that arguments for and against OS highlighted at the height of the pandemic—which were largely well-established before it ( Benson Marshall et al., 2024)—had not substantially changed. The benefits—scientific, economic, and social—were widely recognised. As we comment in Benson Marshall et al. (2024), it perhaps should not surprise us that these arguments had not substantially changed – if they were fundamentally sound before pandemic, we would not expect them to change, but perhaps would expect the pandemic to illustrate them, as some of our participants clearly did. Equally, it clear that concerns about OS remained, particularly those relating to quality, misinformation, and equity. These issues are discussed briefly in this section.

Significantly, however, our analysis suggested that many of these focuses of debate were manifestations of underlying systemic challenges present within the research environment, which were evident with varying degrees of explicitness or implicitness in interviewees’ remarks, and which many participants now saw as affecting moves in the direction of greater openness and needing to be addressed for OS to become more widely adopted. While these issues were not new, the pandemic served to illuminate them and clarify their significance, demonstrating how particular aspects of the research and publishing processes had been affected by the crisis. Four systemic issues were made evident in the data: the question of the scope of OS itself; regional variations in how OS is being enacted; the relationship between OS and fundamental questions of the way in which science should be done; and the importance of addressing incentives and reward structures within research. We discuss those systemic issues in the sections that follow.

Of the issues brought into particular focus by the experience of the pandemic, preprinting remained a subject of significant interest, having gained wider acceptance among academic communities during the medical emergency ( Brierley et al., 2021; Callaway, 2020; Coates, 2021) and having become a focus of some of the major arguments for and against openness in general ( Benson Marshall et al., 2024; Koerth, 2021; Yan, 2020). One interviewee believed preprints “completely changed the dialogue” in how findings were reported during the pandemic. They influenced policy, as demonstrated in their extensive use by WHO (reported to us in detail in our interviews), and directly impacted medical practices. However, there were also signs that preprinting seemed to be plateauing, or at least returning to pre-pandemic trend lines ( Biesenbender et al., 2024). Despite the benefits, some interviewees raised concerns about a lack of awareness among academics, the media, and the public regarding the role and function of preprints. There were also significant concerns about misinformation ostensibly linked to academic work that had not been peer reviewed.

Alongside preprinting, data sharing and OD were frequently highlighted as crucial developments in the pandemic response. Some interviewees even suggested that the availability of reliable data was more important than access to research outputs based on that data, allowing scientific work to be done faster and more productively, driving change and insight as well as facilitating understanding of the output. However, while OD was acknowledged as useful in understanding the spread of COVID-19, there was some scepticism regarding its practical impact, with one interviewee claiming that they saw no acceleration of knowledge leading to interventions or better diagnostics based on OD or OS in general. There were also concerns over consistency, format, disciplinary preferences, ownership, and curation of data. One new area of discussion was open methods, which were seen as valuable in enabling reuse of data.

While many interviewees agreed that OS in general positively impacted the pandemic response, there was variation in the perceived extent of its influence. Some saw the pandemic as a “stress test”: an unprecedented opportunity to examine the efficacy of OS principles in a real-world, high-stakes environment, and to demonstrate the benefits of increased speed and collaboration. This had particular influence on policy development, being a “useful catalyst” for funders to formalise OA policy and mandates (Damian Pattinson, eLife).

P9 went further in suggesting that “the nature of the way open science is conceived has changed” in fundamental ways as a result of the pandemic acting as a “concrete use case” and making the benefits of OS clearer to scientists and the public. They went on to state that:

“the broader trends that were accelerated during COVID are definitely here to stay and all I see now is the momentum for open science really growing around the world … I don’t see it slowing down any time soon. I think it will be the new normal and in five years everything will be open.”

However, the initial hype surrounding the role and development of OS during the pandemic ( Callaway, 2020; Shearer et al., 2020; Taraborelli, 2020) may have been overstated. The pandemic had not resulted in the rapid systemic shift towards greater openness that many of its advocates had hoped for in the early stages of the pandemic. The analysis that follows goes some way to explaining some of the issues associated with that stasis. Some interviewees expressed uncertainty about the progress made, noting only incremental change in the scientific environment: “we’ve moved a little bit further and it has shifted some people, but I think we’ve largely slid back to where we were before” (Rebecca Lawrence, F1000). There was also some scepticism about the role of OS. Publishing consultant Kent Anderson felt that OS had played a “hugely confusing and counterproductive” role, and that its impact on the pandemic response was overestimated. He instead cited the long history of previous vaccine research as being more impactful than any developments in OS.

### 4.2 What constitutes open science?

As noted, the first fundamental and systemic issue that emerged from our interviews was the question of what constitutes OS and what that means for policy and practice. The publication of the UNESCO Recommendation on Open Science (2021) brought to the foreground ongoing discussions about the scope of OS and was seen as significant by many of our participants, especially as it presents a broad conceptualisation of the ideas and values of OS. In addition to concepts of “open scientific knowledge” and “open science infrastructures” found in many European and North American discussions of OS, the Recommendation emphasised two other pillars of OS: “open engagement of societal actors” and “open dialogue with other knowledge systems”. This wide interpretation of the scope of OS provided a focus for discussion with interviewees, some of whom were more receptive to it than others. We had actively recruited participants who we expected to provide different perspectives these issues, which proved to be case, although the heterogeneity of our sample was limited in some areas. For example, worked in science communication, as one way to address the topic of “open engagement of societal actors”, representation of this element was ultimately limited. However, in other areas, such as publishers and infrastructure providers, we heard a range of views.

Several interviewees were involved in or were able to observe the process of developing the UNESCO Recommendation, which began in 2019 but was largely undertaken in the context of the COVID-19 pandemic. These participants reported that this distinct context not only shaped the Recommendation’s content, but also mobilised broader engagement with its development, making the process more equitable and inclusive by enabling and encouraging participation from a wider range of individuals and groups. This broader engagement, participants claimed, allowed for connections with societal actors and other knowledge systems to be brought forward as central consideration of OS. While these elements have long been a core theoretical aspect of the academic debate around openness ( Bull, 2016; Chan, 2002; Fecher & Friesike, 2014; Grahe et al., 2020; Pinfield, 2024; Stracke, 2020; Willinsky, 2006), especially in Latin America, the level of focus on these issues by different actors and groups, especially in terms of their practices, has varied. In some quarters more instrumental arguments for OS, with emphasis on benefits primarily for the scientific community (‘making science more efficient’), have been foregrounded.

Similarly, participants commented on how the Recommendation has explicitly highlighted the importance of equity in debates about OS, albeit debates which focus on international, rather than social, equity. They felt that the equal status given to equity-related aspects of OS in definitions such as UNESCO’s are giving the topic greater attention and allowing these elements to be brought in at policy level, although this conflicts somewhat with the findings of Chtena et al. (2023). This shift in discussions about OS—what might be called an *equity turn in OS*—aligns with broader societal trends towards equity, diversity, and inclusion, and was another focus of discussion in the interviews, eliciting a range of responses. One interviewee explained it as follows:

“equity, fairness, transparency, visibility, the fact that all these things were taken away from us during the pandemic as it were, by working in closed groups, not [being] able to discuss as freely as we had done before, underlined their importance in a post-pandemic world … And because it was patently obvious that different countries and different communities in different countries were coping better or not so well with the pandemic, the issue of equity and fairness and justice was really a driving force behind the [participant’s institution’s] statement … that open science was the way forward in a post-pandemic world.” (P6)

Some participants saw the equity turn as very important, perhaps crucial, to the future of OS. However, some were less concerned with it as a focus for OS work, preferring to concentrate instead on open knowledge and infrastructure, which they saw as core to OS. For example, Susanna-Assunta Sansone of the University of Oxford described the expanded definition of OS in the UNESCO Recommendation as “not my definition of open science.” She explained that her work addresses issues around tools, resources, and infrastructures—the technical side of open knowledge and open infrastructure, rather than discussion around who creates or accesses it. Others were less direct about stating a different set of priorities, but still implied that their focus was more on the aspects of OA and OD relating to access to outputs and improving open infrastructures, which for many had traditionally been prioritised in the way the case for OS is made and in how OS is implemented.

Many participants readily signalled their acceptance of all the pillars of the UNESCO Recommendation in general terms, as in this quote from Torsten Reimer, University of Chicago Library: “But ultimately assuming, this is my belief, that research is there to be a greater good for society and humanity overall, then you want that widest level of access and we need to think [about] that beyond academia.” However, while participants generally agreed these aspects were important, there was uncertainty around what they meant in practice: “…these statements [are something] I think almost everyone would subscribe to. The question is really, how can you put this into action?” (P20). This lack of clarity on what the equity turn may look like in practice is something that appears to be a wider challenge, as many policies and guidance documents use the language of equity, but often fall short in providing specific guidelines for its implementation ( Chtena et al., 2023). Another participant warned of the danger of “equity washing”: engaging superficially with equity initiatives without making substantive changes to address systemic inequities (Catriona MacCallum, Wiley). In such a way, there may be a false sense of progress while existing disparities are perpetuated. The underlying issue seemed to be how to bring the various components together in a meaningful and productive way, as described by one participant regarding a conference they had attended:

“there were presentations on the output accessibility and transparency end of things, and there were presentations on the inclusion end of things. But what I thought was missing from that event was really stitching those things together and placing them into meaningful conversation with one another rather than just saying that they’re both good.” (P18)

Several participants noted that OS had been seen as a Global North interest, and pointed out how Global South countries were sceptical and wary of risks with OS, including exploitation, which they had experienced in the past and continued to be a broader concern. These concerns were reflected in the discussions that led to the drafting of the UNESCO Recommendation:

“[OS] was very much kind of a Northern concept. And the interest from countries from the South was not huge. They were very sceptical about what it means to open their science, contrary to what one would think. They were very sceptical and they found a lot of risks in opening their data and other knowledge because of the past situations because of the possible risk that they could be exploited and they would not be able to access the data because they don’t have the adequate technology.” (P16)

This view linked OS to concerns around what is seen as colonial, helicopter, or parachute research, terms which describe “a situation where researchers from wealthier countries … fly to a developing country (global south), collect data and specimens, fly out, analyse the data and specimens elsewhere, and publish the results with little involvement from local scientists” ( Minasny et al., 2020). Reggie Raju, of University of Cape Town Libraries, stated that Africa was particularly threatened by this: “Africa is a prime area for research, but we don’t have access to that research. We can’t afford it.”

Several interviewees, including some who were involved in the development of the UNESCO Recommendation or its implementation, chose to discuss the pillar of “open dialogue with other knowledge systems” and its specific reference to Indigenous knowledge systems. The involvement of Indigenous communities in this development was itself contentious, with some participants reporting that some community representatives had been reluctant to be involved in the development of the Recommendation. Interviewees stated that these communities felt “othered” in the process due to the terminology used, and perhaps more significantly, that there were substantial concerns about sharing traditional knowledge:

“I was sceptical about this because I was worried about the autonomy of the Indigenous people and this type of knowledge is not exactly desperate to be a part of the traditional knowledge … it’s not that you’re involving them. You are forcing them to share a knowledge, with the infrastructures and the system that you decide that is the adequate platform for open science. And who is going to govern that? And if the authority is in their hands they should have the authority to decide… Indigenous knowledge is not a peripheral knowledge … It is created by a community. It is a collective type of knowledge … There is a collective authorship of the Indigenous communities. How is this going to be recognised?” (Fernanda Beigel, Universidad de Cuyo, Argentina)

The integration of Indigenous and Western perspectives is often emphasised in discussions of OS, including the UNESCO Recommendation, as a means of decolonising and enriching scientific knowledge ( Dutta et al., 2021; Lui et al., 2021; Ngulube, 2023). However, as some of our participants recognised, there is a tension between making Indigenous knowledges more widely accessible and protecting the cultural practices and beliefs often associated with them ( Ravindran, 2024). There is also the difficult problem of how different epistemic systems can or should be integrated ( Pinfield, 2024; Santos, 2014). While there is a push for greater accessibility and acceptance of Indigenous knowledges, there is also a need to respect Indigenous sovereignty over their own data and knowledge ( Ravindran, 2024; Traynor et al., 2019), as espoused in the CARE principles ( Global Indigenous Data Alliance, 2023; UNESCO, 2021). At the same time, there is also scepticism about whether Indigenous knowledges can or should be seen as equivalent or even relatable to science ( Basken, 2023). This highlights the complexities and controversies of applying OS principles to Indigenous knowledges, which often operate within radically different cultural and epistemological frameworks.

Several interviewees argued this discussion was important within OS, suggesting that Indigenous knowledge should be recorded, preserved, and made accessible to the communities who own it, and potentially other communities worldwide. This is a complex challenge, however, and several participants explored ways to address it. Indigenous knowledges often do not have the same formal expression or codification as science, so documenting them and preserving the record is likely to be challenging. However, the concern then is around curating the knowledge only, or primarily, for the communities who own it, to avoid exploitation by other parties. Indeed, even framing the issue in this way might unintentionally imply an existing deficiency in Indigenous knowledges. Similarly, participants noted that distinguishing ‘Indigenous knowledge’ from ‘Western knowledge’, as is often done, can suggest the former is somehow inferior or irrational. Indigenous groups in Brazil, for example, take a different view of education and expertise, and these concepts are interpreted and practised differently than in other geographic and cultural contexts (Luisa Massarani, Sci-Dev.net). Engagement was seen as key to addressing some of these issues, including talking “with, not about, [Indigenous peoples’] concerns … concern alone is not enough. You also need to actually engage and make sure that the community has a positive benefit as an outcome of their engagement” (Jo Havemann, AfricArxiv/Access 2 Perspectives).

### 4.3 Global and regional variations

As well as having a complex, context-specific relationship with other knowledge systems and practices, OS itself was seen by many of our participants to be subject to significant regional variations in understandings, priorities, and implementation strategies—something that might be captured by the concept of *context-specific openness.*

There was an implicit assumption shared by some participants from the Global South that awareness of OS issues amongst key actors was greater than in their own contexts. However, some suggested that there was growing awareness and adoption of OS in their geographic areas, albeit not without resistance or scepticism in some cases. For example, Moumita Koley, of Indian Institute of Science, stated that “people are taking openness more seriously” in India, although this is driven by norms set by those outside research performing organisations, such as funders or the media. However, there seemed to be little incentive to adopt open practices in other countries, such as China. For many participants, access to scientific knowledge and infrastructure remained a major challenge in much of the Global South. The high costs of article-processing charges (APCs) as a way of paying for open access continued to cause significant concern to participants in Latin America, who noted increasing resistance to the APC system globally—for example, in the resignations of several journal editorial boards ( open-access.network, 2023; Retraction Watch, 2023; Sanderson, 2023). Concerns over commercialisation featured strongly in these discussions, with APCs described as “a pyrrhic victory” that ultimately undermined the core principles of OS (Fernanda Beigel).

Adoption of particular forms of openness, such as preprinting, that were prominent during the pandemic were also perceived differently in various regions. In China, despite some ostensible official support for posting preprints, they were still seen as unreliable or less valuable than peer reviewed research: “if you publish with preprints, your results will be difficult or even impossible to have credits for your career” (P24). Their effect on the pandemic in the MENA region was described as “a mess” and “catastrophic”, particularly regarding their use by the media (P5). P5 elaborated that “science journalism became a mainstream journalism”, and, as a result, journalists without scientific background used preprints incorrectly, contributing to dangerous misinformation and medicine shortages. Most specialised science media in that region subsequently implemented policies against covering preprints.

The quality of preprint research was a concern in various settings, and perceptions of quality differed depending on the context. For example, in the African context, Reggie Raju described how the University of Cape Town and the developing national South African research repository chose not to include preprints because of concerns over their quality and subsequent perceptions of African research:

“coming from Africa I have always felt that we needed to work doubly hard to prove ourselves. And then having poor or wrong research go out … makes Africa worse as a continent in terms of research. The same mistakes can be made by the Global North because we say, ‘Ah it’s a mistake, it’s okay,’ but [when] it comes here, they say, ‘Ah, you see, it’s Africa and it’s poor research.’ So we have to work that much harder to prove ourselves … we will not put preprints in that national repository because it just undermines the quality of the research that comes out of South Africa and the continent.”

He related these concerns to the balance of incentives for researchers regarding the pressure to publish (including on preprint servers) versus the quality and integrity of their research, noting: “I see the kind of chances people take to get published.” It seems that the balance of incentives may work differently in different contexts, something we discuss in more detail below.

Adoption of open practices, and associated incentives and rewards, are often influenced by the local policy environment, and this varied across different geographical contexts. For example, Raju described the Namibian government as having “bought into openness”. Many European interviewees assumed a relatively robust OA policy environment, promoting OS adoption. In the USA, participants described the memorandum on ‘Ensuring Free, Immediate, and Equitable Access to Federally Funded Research’ ( Nelson, 2022) as a key policy development, clearly given momentum by the pandemic. However, it proved surprisingly difficult to obtain information about the memo, its creation and implementation, in our research. Interviewees with insight into the USA’s political landscape implied that there were doubts about whether there was enough political will to ensure it would be fully implemented.

Participants from China reported that current national science policy concerns were largely focused on research integrity. This created an ambiguous relationship with openness, with a policy adopted in late 2023 discouraging scientists in China from sharing their findings with the public before peer review. Paradoxically, however, the policy allowed researchers to post preprints, which—despite their publicly available nature—were considered part of scientific (rather than public) discourse ( Ministry of Science and Technology, 2023). Interviews also pointed to a trend towards greater self-reliance in Chinese scientific policy following the pandemic, with increased emphasis on the national benefits of Chinese research and reduced emphasis on openness, which is by definition focused on global sharing. This is consistent with evidence of growing “scientific nationalism” in China reported in literature coinciding with, and partly in response to, the pandemic ( Mallapaty, 2021, 2023, 2024). This is illustrated in the 14 ^th^ Five-Year Plan, which outlines China’s strategy for scientific and technological self-reliance, driven by tensions with Western nations. This shift aims to strengthen domestic research capabilities and reduce reliance on foreign technologies ( Mallapaty, 2021, 2023, 2024). Alongside this, one interviewee from China reported that Chinese scientists were being excluded from international data sharing platforms. This is notable, considering China’s increasing restrictions on foreign access to its own data ( Bouey, 2021; Lewis, 2023; MacDougall, 2023). These factors often limited the ability of researchers in China to engage in international collaborations, potentially hindering the global cooperation that was seen as vital for OS.

Against such a varied background of geographical and cultural contexts, the concept of context-specific openness becomes important as a way to highlight tensions between the global and the local. Several participants emphasised the substantial challenges involved in translating policy into practice, for example in evaluating the value of the UNESCO Recommendation, as

“[bringing] all the concerns from different parts of the world together in one movement … [it] doesn’t really recommend anything new, what it does do is bring unification in terms of the approaches so that we’re all agreeing that we will move in this direction, recognising that some of the issues in the Recommendation apply to us while others apply to other countries, say outside Europe.” (P6)

However, while the Recommendation begins to address issues around implementing OS in low- and middle-income countries, it can nonetheless be “overwhelming” for policymakers in those countries to implement in terms of the number of different agendas under the OS umbrella. This was the view of P9, who advocated a tailored, country-specific approach, emphasising the importance of each country deciding their own priorities, selecting the most appropriate OS initiatives to implement based on their specific needs and capacities, and adopting a “staggered approach” to make the process more manageable. What becomes clear from our data is that OS is not monolithic phenomenon based on a universally-shared idealised form, but rather as a pluralistic landscape of possibilities which can only be navigated through contextually-grounded responses to local infrastructures, incentives and epistemic cultures.

Discussion of context-specific openness prompted further consideration of broader issues around Western democratic norms and their influence on OS philosophy. One participant, in discussing the Middle Eastern context, observed that the very concept of openness was viewed quite differently in non-democratic countries—that OS by definition requires a minimum level of democracy that does not exist everywhere. Openness is typically framed as desirable because it adheres to Western liberal democratic norms, or is at least framed in ways that relate to liberal concerns, such as transparency, accountability, and efficiency. As a participant in Latin America noted, “society is asking, we are paying for this [research] and what is the benefit for us?” (Fernanda Beigel). A participant from a non-democratic country suggested that such a question would not arise in non-democratic contexts, which may prioritise different values and governance structures. For example, it was suggested that countries such as Egypt should look to examples of OS outside Western liberal democracies; a Western approach might not fit with specific local infrastructure and priorities.

These concerns fed into broader questions about the relationship between liberal democracy and science itself. OS agendas have often been linked to the conceptualisation of science and “open society” liberalism ( Merton, 1942; Popper, 1945), in which successful science (involving freedom of inquiry and self-governance by the scientific community) is more likely to be successfully pursued within a liberal democratic context. The success of the Chinese science system does, however, seem to challenge this model ( Wagner, 2024), although arguably the Chinese science system tends to be focused on specific areas of science, and addressing particular questions, which avoid political controversies. In this sense, these discussions about the role of OS in diverse national and political contexts raised a larger question of how science itself should be practised.

### 4.4 How should science be done?

Underlying many of our participants’ remarks were differing assumptions on the fundamental issue of how science should be done. The discussion around preprints, for example, often centred on the way preprinting challenges traditional scientific practices by sharing preliminary findings before peer review, opening them up at an early stage to public scrutiny and discussion. The debate is at base about how the scientific process should best be carried out, and in particular, the extent to which scientific discussion should be carried out in public—a question that the pandemic brought into sharp relief.

Preprints were just one example of how participants’ varying attitudes to openness reflected different perspectives on the fundamental question of the best way to do science. Some participants argued that openness is an inherent part of good science, characterising OS as “how to do science right” (Nokuthula Mchunu, African Open Science Platform). Jo Havemann framed the OS principles as “good scientific practice and nothing more, nothing less … in all the trainings, including scientific writing and publishing, I’ve always said the baseline was open science principles … the open science debate reminds us of why we’re doing the research in the first place.” This approach often aligns with the Mertonian scientific norm of *communism* ( Merton, 1942)—the collective ownership of scientific findings—in that participants saw sharing research within the scientific community as essential for promoting the kind of debate and collaboration that make scientific work possible:

“when science is being done in private, just for satisfying somebody’s curiosity, but it is not shared … it cannot be built upon, it cannot inform anybody… Science is [a] social endeavour and that requires both scientists to share what they have discovered and for others to critique and improve and build upon that discovery. So open science, openness isn’t just a ‘nice to have’ thing. It’s an essential feature of how science is done, always.” (Bodo Stern)

This process of critique and improvement corresponds to another Mertonian norm of *organised scepticism* ( Merton, 1942), which emphasises the importance of critical evaluation of findings and methods before their acceptance by the scientific community. One interviewee observed that “science is filled with not knowing … going backwards and forwards and that this is kind of a normal procedure in science” (P20). OS arguably makes such critical evaluation within scientific systems more easily possible.

However, the extent to which it was advisable to expose the “backwards and forwards” of science beyond the science community was disputed. One participant stated that scientists should be encouraged to “let different publics, both within the science system and outside the science system, know [when there is] contestation around different knowledge systems coming into contact with one another, and not communicate that something is fixed or broken, but that [there is] a multiplicity of ways that people reason about the world” (P18). Bodo Stern noted that science conducted in a closed fashion attempts to “sanitise” the true nature of science, which is incremental and error-prone.

However, other participants expressed that exposing the iterative and self-correcting nature of science can compromise public trust in science, particularly when science is under intense scrutiny and has significant implications, as was the case during the pandemic. One approach to address this might then be to shield scientific processes involving doubt and contestation from the public gaze by conducting the science in private, in order to minimise the potential for stress, misinformation, and misunderstanding. This is reflected in comments such as Kent Anderson’s conceptualisation of the public in relation to scientists: “We’re the crowd, they’re the players.” Such comments suggest a clear division between the public and scientists, with the latter trusted to get on with the science. In this view, scientific discussion and debate should take place exclusively within the scientific community—among the “players” of the game—before the products of science are released to “the crowd”, for example, in the form of vaccines in the case of COVID-19. Relatedly, science should be communicated to the public in a clear way, without allowing an unnecessary plurality of voices and interpretations.

In contrast, other participants strongly advocated public involvement in science. For example, one interviewee who worked in publishing stated that it is unhelpful to dismiss public concerns about science as irrelevant or beyond the public’s understanding, particularly in the current political climate. A more effective strategy would involve openness, transparency, and proactive engagement with the public, and OS partly provided such transparency. Along similar lines, another participant working in science policy contended that “we still put science in this ivory tower with very smart people doing very smart things that nobody else understands”, criticising the isolation of science and emphasising the need to make it more, not less, accessible (P16). However, to make this approach effective, they argued, intermediaries who can bridge the gap between science and society are essential.

Participants related this debate to questions of how scientists should view their relationship with wider society, questions which again became more acute during the pandemic: “I think that a very important thing is that scientists understand that they are included in society. I think that scientists are still thinking about [themselves] as something different [from] society” (Luisa Massarani). This quote implies that scientists often see themselves as “other” than society, in line with Anderson’s view of the “players” being distinct from the “crowd”. In this view, the importance of sharing science is restricted to the scientific community and not the wider public. It is also counter to the view of OS as encompassing societal engagement expressed in the UNESCO Recommendation. This then gives rise to the question of how science should be communicated, both among the academic community and to the public. Some interviewees commented that scientists often lack the skills as well as the incentives to communicate their work to the public effectively: “This dialogue between science, policy, society—there have to be people who are able to articulate that conversation a bit better … I’m not sure if we should ask our scientists to be more open communicators or we should form another group who is able to do that, including scientific journalists.” (P16)

A repeated topic of discussion was the related issue of public trust in science, and how the pandemic and the increased openness it introduced had affected this, both positively and negatively. These discussions again connected to the question of how science should be done, particularly the issue of whether transparency helps or hinders public trust. Interviewees saw public loss of trust in science as a major challenge, as observed by Torsten Reimer: “I think the pandemic has accelerated that [loss of trust, with] some parts of society decoupling themselves from the consensus that science and research is something to be trusted.” While the issue of transparency and trust is not entirely new, it has gained increased attention due to the successes and failures observed during the pandemic.

Some of the discussion around public trust touched upon the underlying issue of scientific literacy—put briefly, the public’s ability to read, understand, and engage with science ( Laugksch, 2000; Miller, 1992; Wynne, 1991)—as well as their desire to do so. Some interviewees appeared sceptical of the public’s ability to critically evaluate scientific information, or felt that public involvement was unhelpful and complicated matters. However, others viewed the public’s abilities more favourably, such as Jo McEntyre of the European Bioinformatics Institute, who stated, “I never underestimate the public … if you’re motivated to read scientific papers, you can actually do it … so I think it’s good to have these things in the public domain … it’s just about the tools to understand.” Participants alluded to a need to move beyond scientific literacy related to scientific facts, and to further consider the public’s understanding of the internal processes of science ( Millar & Wynne, 1988), such as the nature of peer review or the potential for any research finding to be disproven. As one participant explained in their discussion of balancing transparency with the risk of misinformation:

“there were some missed opportunities in helping people outside the science system to understand when science was working exactly as it was supposed to, things that perhaps looked worrisome but that were in fact a very healthy process of science establishing which claims would last and which claims would not … openness and transparency are a key part of the solution there, but they [should be] scaffolded by synthesis, by clear and persuasive analysis … it’s not enough to just do a data dump and to say we’re really being transparent, but it’s about giving people the tools to make sense of what is being made transparent.” (P18)

Some of the participants favouring OS took on an optimistic perspective of such public transparency, summarised by Nokuthula Mchunu: “I think if we really continue doing science in an open way, we will kind of be able to bring society back into it.”

### 4.5 Reforming incentives and rewards structures in science

Participants saw the pandemic as revealing the powerful role of traditional incentive and reward structures, exposing a certain inertia in scientific systems. While the crisis prompted temporary shifts in norms, these do not appear to have created a more permanent shift towards open practices. Several interviewees emphasised the need to change attitudes around what is considered valuable or important in science in order to transform the way research is conducted: “we need to change what counts” (Bodo Stern). Participants advocated for a change in scientific culture, arguing that the competition to claim priority for scientific findings and publish in prestigious journals should no longer overshadow other values such as quality, integrity, and openness. This line of argument often led back to the fundamental question of why science is done in the first place: “why are we doing the things we do? How do we do knowledge generation as a global good?” (Nokuthula Mchunu). The connection to societal good was echoed by P20: “I think this requires cultural change. Because in the end the question is, what is this all about? Is this about getting a publication out, or is it about this kind of knowledge creation and making knowledge known to the community and beyond?” The question of culture and attitudes is, of course, affected by the many different contexts in which (open) science is conducted and disseminated worldwide. Some contexts are characterised by a greater or lesser awareness and positive inclination towards OS, as we have seen.

The discussion of how science should be done, including how open practices should be incorporated in it, led many interviewees to address the shortcomings of the current scientific incentives and rewards systems. Widening acceptance and adoption of openness in science did not lie just in improving open technologies and processes, or in extending open infrastructures, important as those steps were to many participants. Rather, they described a need to reform the broad incentives and reward structures and cultures shaping the attitudes and behaviours of researchers. This would involve changing evaluation systems to incentivise open practices, encouraging a different way of doing science that rewards a set of practices associated with more collaborative and open behaviours. This perspective represents a shift from a focus on technocratic solutions to the OS challenge—improving technologies and processes—to one centred on cultures and reward systems.

Catriona MacCallum advocated reforming the system to place equal importance on the principles of “validity, integrity, trust”, rather than publishing in prestigious venues or securing funding. This was also seen as foundational by publishing consultant David Crotty: “the reality is pretty much everything stems from the funding and career structures of academia. If you want to reform publishing, if you want to reform data sharing, you have to reform those two things”. P9 used the metaphor of “a three-legged stool”, with the first leg representing “policies and research assessment policies and other open access policies, [the second representing] support and infrastructure, and the third one is just the research culture, so those three things have to grow at the same time if you want to be able to sit on the stool without falling off.” Acknowledging the complex nature of research, such reform would involve a wide range of actors, working in concert: “it’s such a systemic problem that affects all the stakeholders and all the stakeholders have to act in some ways in unison in order to move the needle” (Bodo Stern).

However, participants also acknowledged that putting these ideas into practice is extremely complex, and the barriers and challenges are both large and diverse; as noted by Jo Havemann, “it’s messy and it’s massive”, although she also expressed hope that this complex landscape is navigable through collaborative efforts and a commitment to inclusivity. Reforming the incentive system among researchers is also complicated by the diversity of global contexts. For example, as noted in Section 4.3, the balance between the pressure to publish, on the one hand, and the importance of research quality and integrity, on the other hand, and how these relate to academic reputation and productivity expectations, differs depending on the local context, and its policy environment and reward systems. Additionally, participants noted the increasing commercialisation of science and scientific publishing; the future of science, and therefore OS, will be led by “where the money goes” (participant working in publishing). Attempting to fit OS into a pre-existing commercial model, as is currently the case, is unlikely to succeed.

Other challenges still persist in the wider adoption of OS, as discussed more extensively elsewhere in the literature; several were mentioned by participants, which we summarise here. Inadequate funding and misaligned funding priorities can limit the potential of OS initiatives, while APCs and unsustainable business models continue to pose financial burdens to researchers and institutions. Infrastructure limitations, such as insufficient data storage and computational resources, are a particular concern in the Global South. Capacity building remains a significant challenge, with many researchers lacking the necessary skills and training to engage in OS practices. Measuring and monitoring openness is another barrier to OS adoption, as there is no universally accepted framework to assess its implementation and impact. While policy initiatives have grown in recent years, they remain difficult to translate into practice, and are often not sustainable or sufficiently adaptable to different contexts.

## 5. Conclusion

This study aimed to identify which OS debates gained traction among influential actors after the pandemic, and to understand the underlying systemic issues that shape how these influential actors see the development of OS in the emerging post-pandemic context.

Our findings suggest that many issues debated during the pandemic, such as the value of preprinting and data sharing, remained crucial in the post-pandemic context. While preprinting gained wider acceptance, concerns about misinformation and lack of awareness persisted. Open data was seen as crucial for understanding the spread of COVID-19, but there were concerns that data sharing remained limited beyond this specific context. The pandemic continued to be seen as a ‘stress test’ for OS principles, and whilst the initial hype surrounding OS adoption during the crisis may have been overstated, the pandemic was seen to have widened awareness of OS and strengthened OS policy development.

Perhaps most consequentially, our analysis documented that in the emerging post-pandemic context the debates about OS often seemed to reflect issues related to fundamental aspects of science—the nature of science ( *what* constitutes science?), its purpose ( *why* do we do science?), and methods ( *how* should we do science?). This demonstrates that, as seen from the vantage point of experts post-pandemic, the debates about OS are not limited to a concept of OS as a superficial bolt-on to science, but rather involve consideration of fundamentals—the *what, why,* and *how* of science. Questions about the context-specificity of OS, and regional variations in how and to what end openness is enacted, also relate to the *where* of science. All these questions gained prominence during the global emergency, when there was particular intensity around the question of the role of science in society, especially in relation to public trust. The remarkably rapid response of the science community to the pandemic at the same time illustrated where inertia in science systems lies, partly created by established incentives and reward structures.

The resulting discussions around the scope of OS, and the scope and purpose of science more broadly, often coalesced in our data around the discussion of the UNESCO Recommendation on Open Science. Given that the Recommendation was written during the peak of the pandemic, it is hard to disentangle the effect of COVID-19 on the debates about OS from the shape taken by the Recommendation. The pandemic influenced the Recommendation, and the Recommendation has become a focal point of debates for many of the issues related to OS and how OS in turn relates to science more broadly.

Our findings demonstrate that context-specificity is essential to how OS is conceptualised, incentivised, and practised. International recommendations for OS, while valuable, often overlook the importance of context-specific approaches, rendering such universalist approaches to OS unlikely to succeed, and possibly even damaging local and national communities. For historically marginalised communities, such as Indigenous groups and those located in the Global South, context-specificity is especially crucial. More broadly, there was significant variance in the political, medical, and scientific response to the COVID-19 pandemic across geographies, including by communities that have often been excluded from, or been peripheral to, mainstream scientific practices. The Western, liberal democratic, Global North perspective that underpins many OS policies and advocacy efforts often does not translate to other contexts.

The UNESCO Recommendation embraces this idea of diversity of contexts and has helped to give it prominence in the ensuing debates. However, the Recommendation does not address the tension between sharing community knowledge, which is essential for diversifying science, and protecting this knowledge—that is, aligning with Indigenous cultural practices and preventing helicopter science. The pandemic illuminated these existing tensions surrounding community knowledge and inequities within science and scientific communication. Different communities faced varying impacts from the pandemic, and experienced varying impacts on OS practices. This insight fills a gap identified in our previous research relating to the published debates on OS, where there was often an absence of contributions from the Global South ( Benson Marshall et al., 2024). Our current results provide more understanding of different geographical and cultural contexts, viewpoints, and practices around openness during the pandemic, and this in turn foregrounds the importance of considering such diversity in future OS strategy and policy.

While the UNESCO Recommendation appears promising in principle, a common view among participants was that it is challenging to implement in practice, in part because of these culture-specific tensions and in part because of its abstractness. This is significant because participants viewed the Recommendation as highly influential following the COVID-19 pandemic, but often felt overwhelmed by the challenge of knowing how and where to begin to implement it. Another area of disagreement was the relationship between science and the public, often linked to concerns about misinformation, public trust, and the challenges of sharing research in progress, all of which were highlighted during the pandemic. These differing perspectives also extended to fundamental views on how science is done—or should be done in relation to those beyond the science community. Many participants believed that openness is essential for effective science. They advocated for both scientists and the public to have access to the internal workings of science, including ongoing research. Others did not consider openness as a core principle of the practice of science but felt it could be used to share the final, settled products of scientific inquiry. This debate was further intensified by the public health emergency, which brought these questions to the fore.

For those who saw a need to widen acceptance and adoption of open practices, many advocated the necessity of a shift in scientific culture at the same time to emphasise values such as quality, integrity, and openness over the desire to be first to publish or to publish in particular venues. The pandemic demonstrated the value of collaborative efforts and more equitable practices in addressing urgent global health challenges, as well as highlighting concerns around public trust in science. This seems to illustrate the need to reform incentives and reward structures to encourage open practices. Achieving this involves shifting the focus of those advocating and implementing OS from technocratic solutions (emphasising technologies and processes) towards prioritising cultures and value systems.

In particular, to effectively enhance global scientific systems for future large-scale health emergencies, such as pandemics, the focus must extend beyond mere improvements in tools and infrastructure, towards a fundamental reconfiguration of the institutional and geopolitical architectures that govern the operation of scientific openness. This involves addressing issues of equitable access, data sharing, and research collaboration. Ultimately, fostering robust global health security necessitates a shift towards collaborative frameworks and revised governance mechanisms that prioritise collective action over individual state interests. This work requires a concerted effort from various actors, including researchers, institutions, and funders. Such effort is likely to require a reassessment of the commercialisation of science and scientific publishing. It requires engagement with the fundamental question of the purpose of science and its relationship with wider society, issues highlighted by the COVID-19 pandemic and the scientific response to it.

## Data Availability

Harvard Dataverse: “It’s messy and it’s massive”: How has the open science debate developed in the post-COVID era?, https://doi.org/10.7910/DVN/K9QSWD ( Benson Marshall, 2025). Data are available under the terms of the Creative Commons Zero "No rights reserved" data waiver (CC0 1.0 Public domain dedication).
