# Supplementary material for: “It’s messy and it’s massive”: How has the open science debate developed in the post-COVID era?
Source: F1000Res. 2025 Aug 4;14:500. Originally published 2025 May 19. [Version 2] doi: 10.12688/f1000research.162577.2 (PMC12171716; doi:10.12688/f1000research.162577.2)
Supplement: Peer review history

# Reviewer report: Reviewer response for version 2

**DOI:** 10.5256/f1000research.185903.r402368

**Published:** 2025-08-25

**Authors:** Richard Holliman

**Affiliations:** 1 The Open University, Milton Keynes, England, UK

The authors have addressed my comments on the initial version. I recommend acceptance.

Is the work clearly and accurately presented and does it cite the current literature?

Yes

If applicable, is the statistical analysis and its interpretation appropriate?

Not applicable

Are all the source data underlying the results available to ensure full reproducibility?

No source data required

Is the study design appropriate and is the work technically sound?

Yes

Are the conclusions drawn adequately supported by the results?

Partly

Are sufficient details of methods and analysis provided to allow replication by others?

No

Reviewer Expertise:

Engaged research, ethics and epistemology.

I confirm that I have read this submission and believe that I have an appropriate level of expertise to confirm that it is of an acceptable scientific standard.

# Reviewer report: Reviewer response for version 1

**DOI:** 10.5256/f1000research.178802.r386192

**Published:** 2025-06-20

**Authors:** Richard Holliman

**Affiliations:** 1 The Open University, Milton Keynes, England, UK

**Overview**

 The paper is well written, and referenced effectively. The data is presented appropriately, and the interpretations are defensible. I’ve recommended changes to the paper, but on the understanding that fundamentally the research has been conducted appropriately, and the findings will contribute to knowledge and understanding in relevant fields.

 Having said this, the paper could be strengthened in several ways, principally related to the framing of the research in the introduction, and some details about the methodology and methods. These initial framings and additional detail should strengthen the interpretations.

**Title**

 I’m not convinced that the quote in the title does justice to the key findings from the paper. If this is about the contextualisation of open biomedical sciences within a plurality of research systems in the post-COVID era, make that clear in the title.

**Abstract**

 Whilst the term ‘stakeholder’ is widely used, it “may inadvertently perpetuate colonial narratives and reinforce systemic inequities” (Reed et al., 2024). My advice is to use another term.

**Introduction**

 The key finding from the paper would appear to be the ways that Open Science (OS) is defined within different research systems. This is confused somewhat towards the start of the paper by the authors describing research as a single system.

 Several key questions follow that need to be addressed in the introduction to strengthen the clarity of the paper:

 1) Are the authors arguing that there is one global research system?

 2) What do the authors think constitutes a research system, either globally, nationally, or more contextually?

 3) How does the concept of a research system apply in the context of publicly- and privately-funded research? (This has implications for the arguments about democracy and science later in the paper.)

 4) How does the authors’ conceptualisation of a research system apply in the context of this research, i.e., are they investigating a generic global research system, a ‘scientific’ research system, a ‘biomedical’ research system as defined by those researching aspects of COVID, or something else? (The answer to this question has important implications for the parts of the UNESCO document on ‘Understanding open science’, and to other academic fields approaches to open science.)

 Related to the points about, the authors need to define open science in a general sense, and then apply it in the context of this paper. The UNESCO documentation, e.g. Understanding open science, should be helpful in this respect, but should not be applied without nuance, e.g., engagement with ‘Open Dialogue with Other Knowledge Systems’ requires respect to those ways of coming to know and sharing of knowledge. Traditionally marginalised scholars should not be required, at least not in any simplistic way, to follow the principles and practices of ‘Open Scientific Knowledge’. In this sense, the application of open science principles should be context-dependent, therefore the paper needs to acknowledge this in the interpretation of the findings.

 Being more precise about the initial scope of the study will support the interpretations and conclusions, which are generally sound if a little simplistic in places. Put simply, a greater focus at the start will help to explain why changes to cultures of research are challenging, because you don’t have to change one system or one culture, you have to change lots of sometimes interdependent and sometimes independent systems and cultures.

 The authors focus on the COVID and post-COVID contexts. It would be interesting to have another paragraph on the lessons learned for open science from previous related epidemics, e.g. SARS (from 2002) and MERS (from 2012). Were the foundations for open science in this broad biomedical field laid down during these earlier epidemics? Did any of these practices endure and inform the global pandemic? This could also link to another core purpose of this paper, i.e. to inform the process of ‘fixing the roof while the sun shines’. There will be another pandemic; the key question for the authors is, ‘how could this paper contribute to how we anticipate and prepare the ground for open science and open knowledge ecosystems to respond?’.

**Methodology**

 The interviewee sample addresses some of the aspects of the UNESCO document on understanding open science, most obviously Open Science Structures and Open Science Infrastructures. It’s much less obvious who is being asked to speak to the other two elements Open Engagement of Societal Actors and Open Dialogue with other Knowledge Systems. In this sense, is this paper about the full range of open science activities as defined by the UNESCO documentation, or more convincingly, a sub-set thereof?

 There were a list of core questions for interviewees; these should be included in the paper or the supplementary materials.

 Did you define open science to the interviewees, or give them access to any of the UNESCO documentation?

 The data analysis section is very brief and emphasises inductive analysis. Were there no research objectives or research questions to guide the selection of interview questions. (The research objectives or research questions should be included in the paper.) Were any of the 120 codes informed by previous theory or findings, or was the analysis purely inductive? How many themes were derived, and how many of them are presented, in the paper? Put simply, this section needs more detail.

**Findings and discussion**

 “You’ve interviewed publishers; how many of them work for profit-making companies, and how many for ‘free-to- **write**, free-to-read’ publishers?”

 You’ve interviewed journalists; the authors should be cautious about generalising this sample to ‘the media’.

**Conclusions**

 There is no one global open research system. Rather, the ways that open science plays out in operational terms is contingent on context.

 What does it mean to be an open scientist, or a contributor to open science? I think the answer will be defined by the context. Incentivising open science requires multiple interventions across research systems, each of which needs to identify and reward what is aspirational in that context. The take-home message could be the exploration of what different actors in research systems across the globe aspire to be, or to realise through their work.

**References**

 (Refer to 1 )

Is the work clearly and accurately presented and does it cite the current literature?

Yes

If applicable, is the statistical analysis and its interpretation appropriate?

Not applicable

Are all the source data underlying the results available to ensure full reproducibility?

No source data required

Is the study design appropriate and is the work technically sound?

Yes

Are the conclusions drawn adequately supported by the results?

Partly

Are sufficient details of methods and analysis provided to allow replication by others?

No

Reviewer Expertise:

Engaged research, ethics and epistemology.

I confirm that I have read this submission and believe that I have an appropriate level of expertise to confirm that it is of an acceptable scientific standard, however I have significant reservations, as outlined above.

## References

[1] : Reimagining the language of engagement in a post-stakeholder world. *Sustainability Science* .2024;19(4) : 10.1007/s11625-024-01496-4 1481-1490 10.1007/s11625-024-01496-4

# Response

**Published:** 2025-07-30

**Authors:** Melanie T Benson Marshall

*(a) I’m not convinced that the quote in the title does justice to the key findings from the paper. If this is about the contextualisation of open biomedical sciences within a plurality of research systems in the post-COVID era, make that clear in the title.*

 The quotation was from a participant, discussing challenges around putting into practice the various suggestions to improve research systems and cultures, which we feel encapsulates some of the key findings of the paper. The quotation in particular and the paper in general are about OS in a wider sense, not confined to one particular field, hence discussion of wider points relating to definitions of OS and incentive systems.

*(b) Whilst the term ‘stakeholder’ is widely used, it “may inadvertently perpetuate colonial narratives and reinforce systemic inequities” (Reed et al., 2024). My advice is to use another term.*

 We note the recommendation not to use "stakeholder" based on the argument in Reed et al., (2024), of which the reviewer is one of the co-authors. In our authorial team, we have differing views of how persuasive we find the argument of Reed et al., and we note that the UNESCO Recommendation on Open Science itself makes frequent use of the word in the Recommendation itself and supporting documents. The term is used there, and has often been used elsewhere as an inclusive one – an attempt to involve a wider range of people and groups in a conversation than might otherwise be the case. That is certainly the case in stakeholder theory. However, as we make only infrequent use of the term in our paper, we have chosen to replace it with alternatives, except when quoting from our participants (many of whom used it, some frequently) or other publications.

*(c) The key finding from the paper would appear to be the ways that Open Science (OS) is defined within different research systems. This is confused somewhat towards the start of the paper by the authors describing research as a single system.* *Several key questions follow that need to be addressed in the introduction to strengthen the clarity of the paper:*

*1) Are the authors arguing that there is one global research system?*

*2) What do the authors think constitutes a research system, either globally, nationally, or more contextually?*

*3) How does the concept of a research system apply in the context of publicly- and privately-funded research? (This has implications for the arguments about democracy and science later in the paper.)*

*4) How does the authors’ conceptualisation of a research system apply in the context of this research, i.e., are they investigating a generic global research system, a ‘scientific’ research system, a ‘biomedical’ research system as defined by those researching aspects of COVID, or something else? (The answer to this question has important implications for the parts of the UNESCO document on ‘Understanding open science’, and to other academic fields approaches to open science.)*

 We appreciate the opportunity to clarify this. In the introduction, we establish a context that gives us space to discuss just such definitions; we do not wish to close off further discussion too early.

 We note that it is common in the literature to use terminology like “the global science system” (e.g. Marginson, 2022: https://www.tandfonline.com/doi/full/10.1080/03075079.2021.1942822). However, this kind of terminology does not imply a homogeneous system but rather a “complex eco-system", encompassing a number of connected national systems, disciplinary communities and other related components. We do not see what we are describing to be one single ‘thing’ and indeed some of the key points we make about the multi-dimensionality of OS and its different geographical instantiations make that point. We have edited the manuscript to reflect this and clarify at each instance of referring to “the research system” or similar.

*(d) Related to the points about, the authors need to define open science in a general sense, and then apply it in the context of this paper. The UNESCO documentation, e.g. Understanding open science, should be helpful in this respect, but should not be applied without nuance, e.g., engagement with ‘Open Dialogue with Other Knowledge Systems’ requires respect to those ways of coming to know and sharing of knowledge. Traditionally marginalised scholars should not be required, at least not in any simplistic way, to follow the principles and practices of ‘Open Scientific Knowledge’. In this sense, the application of open science principles should be context-dependent, therefore the paper needs to acknowledge this in the interpretation of the findings.*

 We have added a working definition of OS to the introduction and further elaborated in the literature review: Rather than approaching open science as a singular, universally applicable model, we understand it as a contested and evolving set of policies, institutions, and practices—shaped by national policy environments, infrastructural conditions, disciplinary norms, and positionalities within the global knowledge economy. We analyse how certain influential actors conceptualise and negotiate OS across a fragmented, pluralistic research landscape, treating these conceptualisations not as deviations from some ideal model, but as contextually grounded responses to local infrastructures, incentives, and epistemic cultures.

*(e) The authors focus on the COVID and post-COVID contexts. It would be interesting to have another paragraph on the lessons learned for open science from previous related epidemics, e.g. SARS (from 2002) and MERS (from 2012). Were the foundations for open science in this broad biomedical field laid down during these earlier epidemics? Did any of these practices endure and inform the global pandemic? This could also link to another core purpose of this paper, i.e. to inform the process of ‘fixing the roof while the sun shines’. There will be another pandemic; the key question for the authors is, ‘how could this paper contribute to how we anticipate and prepare the ground for open science and open knowledge ecosystems to respond?’.*

 The context of previous epidemics is discussed in the first paper from this research (Benson Marshall et al., 2024), as referenced in the current paper. We did not want to repeat that discussion. The current paper surfaces the structural and cultural barriers that constrained OS during COVID (incentives, uneven infrastructures, epistemic hierarchies). It shows that, despite the exceptional circumstances of COVID-19, many of these structural conditions remained largely unaltered. We highlight these as persistent conditions that will limit OS again unless addressed. We have further indicated, in the conclusion, that future readiness does not hinge on better tools or broader infrastructure alone, but on reconfiguring the institutional and geopolitical architectures within which openness is expected to operate.

*(f) The interviewee sample addresses some of the aspects of the UNESCO document on understanding open science, most obviously Open Science Structures and Open Science Infrastructures. It’s much less obvious who is being asked to speak to the other two elements Open Engagement of Societal Actors and Open Dialogue with other Knowledge Systems. In this sense, is this paper about the full range of open science activities as defined by the UNESCO documentation, or more convincingly, a sub-set thereof?*

 We have edited the paper to make it clearer that we actively recruited (at least some) people working in science communication—i.e., Open Engagement with Societal Actors—but still ultimately had limited or no representation around the fourth element. Interviewees often did not acknowledge these elements as part of OS, and even when prompted, their view of them was sometimes narrow and/or superficial. This is acknowledged as a limitation. Importantly, though, we did not use the UNESCO Recommendation as the basis for the research or sampling method; we did not design the study around the UNESCO pillars, although it was one issue covered in our interviews. What became clear in our analysis, however, was the UNESCO Recommendation was a useful tool to organise our findings.

*(g) There were a list of core questions for interviewees; these should be included in the paper or the supplementary materials.*

 These are included in the interview guide, which is linked in the paper: https://dataverse.harvard.edu/file.xhtml?fileId=10907576&version=4.0

*(h) Did you define open science to the interviewees, or give them access to any of the UNESCO documentation?*

 Per our response to point (c), we did not want to give interviewees a pre-determined notion of OS, or the elements defined in the UNESCO documentation, aiming to keep the discussion around conceptualisation of OS as open as possible initially; part of our aim was to ascertain how they conceived of OS. If they were unfamiliar with the UNESCO Recommendation, we provided them with links to this in the interview.

*(i) The data analysis section is very brief and emphasises inductive analysis. Were there no research objectives or research questions to guide the selection of interview questions. (The research objectives or research questions should be included in the paper.) Were any of the 120 codes informed by previous theory or findings, or was the analysis purely inductive? How many themes were derived, and how many of them are presented, in the paper? Put simply, this section needs more detail.*

 The methodology of this study, including selection of interview questions and derivation of themes, was informed by the first resulting paper (Benson Marshall et al., 2024), which we have now mentioned further in the Methodology section, and which is also discussed in section 4.1. We have also added more detail about the coding processes and overall analysis in 3.2. 

*(j) You’ve interviewed publishers; how many of them work for profit-making companies, and how many for ‘free-to-write, free-to-read’ publishers? You’ve interviewed journalists; the authors should be cautious about generalising this sample to ‘the media’.*

 The publishing landscape is a complex one, and the distinctions often made between commercial publishers, university presses, learned society publishers, and others are often not clear. University presses, for example, vary enormously in size and mission. Learned society publishers similarly vary. Open access publishers may be commercial or not-for-profit, with very different missions and priorities. Our interviewees from publishing organisations did not fit neatly into these distinct categories, reflecting a diverse and evolving publishing landscape. Their affiliations as given in our list of participants make this clear.

 Where we make reference to “the media” in the paper, we do so in relation to participants’ comments in particular and so reflect their perspectives.

# Reviewer report: Reviewer response for version 1

**DOI:** 10.5256/f1000research.178802.r386191

**Published:** 2025-06-16

**Authors:** Diana Lucio-Arias

**Affiliations:** 1 Pontificia Universidad Javeriana, Bogotá, Bogota, Colombia

The article explores, through 33 in-depth interviews, the evolution of the discourse around open science (OS) in the post-pandemic era. As such, it enriches previous findings related to OS, where textual evidence (from blogs, journals, and other sources) was used to examine the debate on OS during the COVID-19 pandemic. The authors focus on issues such as preprints, open data sharing, equity, and the transformation of evaluation frameworks to make OS a central feature of scientific practice. The study also highlights the importance of addressing global inequalities in the research ecosystem, particularly in the Global South, and proposes the concept of context-specific openness, which provides an interesting umbrella to develop country-specific policies to promote open science.

 The work is clearly presented and well-grounded in the current literature, with the authors engaging deeply with existing debates and policy frameworks. The study is both timely and relevant, offering new insights into the evolving discourse around open science. I found the study design appropriate, although geographic diversity among the interviewees should be more clearly addressed, as the geographic representation appears to be skewed toward Europe and North America. Including a table that accounts for the geographic distribution and roles of the interviewees could enhance the interpretation of the results. This would allow readers to better grasp regional nuances and the diversity of professional roles represented in the findings.

 I appreciate that the authors provide access to the interview transcripts, which significantly enhances the transparency of the research. However, more specific details on the steps taken (e.g., how themes were identified and validated) would increase the study's methodological rigor. Additionally, including more information about the coding process and the role of team members in interpreting the data would be beneficial.

 The conclusions to prioritize equity and inclusivity within OS, as well as to emphasize the importance of context-specific and geographically tailored approaches to OS implementation, are adequately supported by the interviews. Suggestions regarding the cultural shifts required to make OS central to scientific practice are insightful and grounded in the data collected.

Is the work clearly and accurately presented and does it cite the current literature?

Yes

If applicable, is the statistical analysis and its interpretation appropriate?

Not applicable

Are all the source data underlying the results available to ensure full reproducibility?

Yes

Is the study design appropriate and is the work technically sound?

Partly

Are the conclusions drawn adequately supported by the results?

Yes

Are sufficient details of methods and analysis provided to allow replication by others?

Partly

Reviewer Expertise:

Research Management, research on research, scientific knowledge generation, circulation and use, bibliometrics, scientometrics

I confirm that I have read this submission and believe that I have an appropriate level of expertise to confirm that it is of an acceptable scientific standard.

# Response

**Published:** 2025-07-30

**Authors:** Melanie T Benson Marshall

*(a) I found the study design appropriate, although geographic diversity among the interviewees should be more clearly addressed, as the geographic representation appears to be skewed toward Europe and North America. Including a table that accounts for the geographic distribution and roles of the interviewees could enhance the interpretation of the results. This would allow readers to better grasp regional nuances and the diversity of professional roles represented in the findings.*

 This is included in the supplementary material at https://dataverse.harvard.edu/file.xhtml?fileId=11022267&version=4.0

*(b) I appreciate that the authors provide access to the interview transcripts, which significantly enhances the transparency of the research. However, more specific details on the steps taken (e.g., how themes were identified and validated) would increase the study's methodological rigor. Additionally, including more information about the coding process and the role of team members in interpreting the data would be beneficial.*

*We have updated 3.2 with more detail about this.*

# Reviewer report: Reviewer response for version 1

**DOI:** 10.5256/f1000research.178802.r386194

**Published:** 2025-06-13

**Authors:** Cameron Neylon

**Affiliations:** 1 Curtin University, Perth, Western Australia, Australia

This paper summarises thoughts and responses surrounding open science policy development and implementation in the light of the COVID-19 pandemic, with a global perspective.

 Its primary thrust is that the issues have not substantially changed, but their context or salience has shifted, and this shift has geographical variation, both "North-South" but also more complex shifts, for instance with uptake of preprints.

 A few things struck me -- and the authors may legitimately feel these are outside the intent of their paper -- with respect to the findings and the participant views.

 One aspect is the narrowness of the apparent framing of open science. I was particularly struck by the apparent lack of mention of access to vaccines as relating to Open Science and/or the tensions relating to IP with respect to vaccine development throughout the pandemic. 

 More broadly I was struck by the way the "usual issues" seemed to be at the centre, including (where named) particular figures taking positions that seem broadly coincident with pre-pandemic statements. I appreciate the paper does point to this. I felt it could benefit from more focus on this (perhaps with relevant comparative quotes? - I appreciate this is a lot of work).

 In general terms the paper reports on the views and statements of respondents and I feel it could benefit from taking a more critical stance. If indeed "not much has changed" despite the world-changing nature of the pandemic, the centering of science as a topic of debate in the proximate policy response, and the strong claims made for the value of open science in delivering outcomes and impacts through the pandemic, then this suggests a potential need for deeper self-examination.

Is the work clearly and accurately presented and does it cite the current literature?

Yes

If applicable, is the statistical analysis and its interpretation appropriate?

Not applicable

Are all the source data underlying the results available to ensure full reproducibility?

Yes

Is the study design appropriate and is the work technically sound?

Yes

Are the conclusions drawn adequately supported by the results?

Yes

Are sufficient details of methods and analysis provided to allow replication by others?

Yes

Reviewer Expertise:

open science, research policy, scholarly publishing

I confirm that I have read this submission and believe that I have an appropriate level of expertise to confirm that it is of an acceptable scientific standard.

# Response

**Published:** 2025-07-30

**Authors:** Melanie T Benson Marshall

*(a) One aspect is the narrowness of the apparent framing of open science. I was particularly struck by the apparent lack of mention of access to vaccines as relating to Open Science and/or the tensions relating to IP with respect to vaccine development throughout the pandemic. More broadly I was struck by the way the "usual issues" seemed to be at the centre, including (where named) particular figures taking positions that seem broadly coincident with pre-pandemic statements. I appreciate the paper does point to this. I felt it could benefit from more focus on this (perhaps with relevant comparative quotes? - I appreciate this is a lot of work).*

 We have edited the introduction to make it clearer that these are the debates that were labeled as being about open science—we do not aim to address all the debates that might be considered to fall under the umbrella of OS if defined very broadly. So, as the reviewer points out, vaccine access might be seen as an OS topic, if OS is considered broadly, and IP related to vaccines more obviously an OS topic. Interestingly, neither was raised by our participants directly.

*(b) In general terms the paper reports on the views and statements of respondents and I feel it could benefit from taking a more critical stance. If indeed "not much has changed" despite the world-changing nature of the pandemic, the centering of science as a topic of debate in the proximate policy response, and the strong claims made for the value of open science in delivering outcomes and impacts through the pandemic, then this suggests a potential need for deeper self-examination.*

 The article attempts to summarise a complex picture in relation to the impact of the pandemic on the OS debate as seen by our participants. There were differing views about what and how much had changed in relation to the pandemic, as described in 4.1. What had not changed was the fundamental arguments used to support OS, although many saw the pandemic as illustrative of these arguments. The pandemic, however, had not led to systemic change in the way that some of its advocates had hoped and we make the point (now sharpened up) that some of the issues discussed in the rest of the article contribute to an explanation of why change has been less far-reaching than OS advocates might have hoped in the early stages of the pandemic. We have added text in 4.1 which we hope makes that clearer. We hope that the nuanced way we have tried to present this will contribute to reflective processes in different communities about how the pandemic has contributed (or not) to their work.
